# An Examination of Parents’ Adverse Childhood Experiences (ACEs) History and Reported Spanking of Their Child: Informing Child Maltreatment Prevention Efforts

**DOI:** 10.3390/ijerph191710580

**Published:** 2022-08-25

**Authors:** Tracie O. Afifi, Samantha Salmon, Ashley Stewart-Tufescu, Tamara Taillieu

**Affiliations:** 1 Departments of Community Health Sciences and Psychiatry, University of Manitoba, Winnipeg, MB R3E 0W3, Canada; 2 Department of Community Health Sciences, University of Manitoba, Winnipeg, MB R3E 0W3, Canada; 3 Faculty of Social Work, University of Manitoba, Winnipeg, MB R3T 2N2, Canada

**Keywords:** spanking, physical punishment, corporal punishment, adverse childhood experiences (ACEs), child maltreatment, child abuse, violence against children, household challenges, peer victimization

## Abstract

The current evidence indicates that spanking is harmful to children’s health and development and should never be used by parents or other caregivers. However, the critical factors that inform effective spanking prevention strategies are still not well understood. The objective of the current study was to determine if a parent’s own adverse childhood experiences (ACEs) history was associated with increased likelihood of reporting their child being spanked at age 10 or younger. Data were drawn from the Well-Being and Experiences Study (the WE Study), a community survey of parents and adolescents from 2017–2018 (*N* = 1000) from Canada. The results indicated that a parent’s own history of physical abuse, emotional abuse, spanking, and household mental illness in childhood were associated with an increased likelihood that their child would have been spanked. These findings indicate that a parent’s ACEs history may be related to how their own child is parented and identify families who may be more likely to rely on spanking. Preventing physical punishment is necessary for healthy child development, reducing the risk of further violence, and upholding children’s rights to protection. Parent’s ACEs history may be an important factor to consider when developing and implementing child maltreatment prevention efforts.

## 1. Introduction

Over the past several decades, many studies have indicated that physical (or corporal) punishment is harmful to children. More specifically, research has consistently shown that physical punishment is associated with an increased likelihood of mental and physical health problems, substance use, injury, aggression, antisocial behaviour, poor cognitive development, poor parent-child relationships, violence later in life in intimate relationships [1,2,3,4,5,6,7,8,9,10,11,12], and demonstrates no benefit to the child [12,13]. Importantly, numerous child-serving professional associations around the world including the American Academy of Pediatrics, the Canadian Pediatric Society, and the World Health Organization (WHO) recommend that parents and caregivers should not use corporal punishment (including hitting and spanking) on children and adolescents [14,15,16,17]. Despite the robust evidence of the related harms and recommendations against its use, preventing physical punishment remains a challenging task and an important public health problem. Prevalence estimates of physical punishment commonly range from 19% to 62.5% depending on the country or region, age of the child, and specific type of punishment [18,19,20,21,22,23,24,25]. At the global level, ending violence against children including physical punishment has been a longstanding goal for the United Nations (UN) Convention on the Rights of the Child (CRC) [26,27] and is included in the UN 2030 Agenda for Sustainable Development indicating a target of eliminating all forms of violence against children (Target 16.2) [28]. As of July 2022, 63 States have enacted laws that fully prohibit corporal punishment of children in all settings including the home environment, and an additional 26 States have indicated a commitment to law reform to achieve a complete ban on corporal punishment [29]. In addition to law reform efforts, which sets a clear standard that no level of violence against children is acceptable, these efforts must be coupled with support services and education that provide parents and caregivers with alternatives to physical punishment that do not put children at risk. Finding new ways to inform efforts to effectively reduce or prevent physical punishment remains a global and public health priority.

One strategy for informing physical punishment prevention efforts may be to better understand how to identify families who may have a greater propensity for the use of spanking. Such strategies can be used to identify those at higher risk and provide opportunities for early intervention and reducing harm. Previous research on the intergenerational transmission of violence may provide some guidance. The intergenerational transmission of violence hypothesis indicates that those who experienced violence in childhood will be at greater risk of continuing the cycle of violence with their own children. A recent meta-analytic study found support for the intergenerational transmission of child maltreatment (i.e., child abuse and neglect) hypothesis, yet with only modest effect sizes noting that parents with a maltreatment history are more likely to have children who experience maltreatment [30]. Importantly, intergenerational transmission of violence may be direct (victim-to-perpetrator) or indirect (victim-to-victim) where the parent with a child maltreatment history may not be the perpetrator of violence [30].

With regard to spanking, it has been found that individuals who were spanked as children are more likely to develop positive attitudes about physical punishment and more likely to spank their own children [31,32,33,34]. Research from our group has found evidence of cross-generational transmission with parents’ agreement that spanking is normative being associated with seven times increased odds that their adolescent children would hold the same normative belief [18]. This work also indicated that parents’ supportive spanking beliefs were related to an increased likelihood that their children had been spanked, as reported by either parents or their adolescents [18]. It is also known that individuals who have experienced child maltreatment are more likely to hold supportive physical punishment beliefs [35] and be more likely to spank their own children [36]. However, inconsistencies do exist in the literature with other findings indicating that feeling threatened or humiliated by parents and experiencing severe physical violence were related to being less likely to hold favorable spanking beliefs [37]. 

Expanding research in this area to include an examination of parents’ experiences of adverse childhood experiences (ACEs) may be useful for informing violence prevention efforts. ACEs research has typically included childhood abuse, neglect, and certain household challenges including household mental health problems, household substance use, parental incarceration, and parental divorce or separation [38]. However, recent research has found empirical evidence for expanding ACEs to include spanking, peer victimization, household gambling problems, parental problems with the law, poverty, and contact with child protective organizations (CPOs) [39]. A previous study did find that maternal experiences of ACEs were associated with an increased likelihood that their own child would experience child maltreatment [40]. Additionally, in a study of teen mothers, a maternal ACEs history was associated with an increased likelihood of physical punishment including spanking, and physical aggression [41]. However, what is currently not well understood is how parents’ history of a wider range of individual ACEs may be related to an increased likelihood of their own children being spanked. One study from our group using representative data from Ontario, Canada indicated that a parents’ childhood experiences of being bullied, slapped/spanked, experiencing sexual abuse, emotional abuse, and exposure to physical intimate partner violence (IPV) were associated with increased odds of youth reports of being spanked or slapped, while parents’ childhood experiences of physical abuse were associated with a decreased likelihood of youth reports of being spanked or slapped [42]. More work is needed in this area with a focus on a broader range of specific ACEs experienced by parents and how this may be related to the intergenerational transmission of violence with their own children’s experiences of spanking. 

Given these findings, several notable gaps in knowledge related to effective physical punishment prevention remain. Understanding these deficiencies in the literature may help to inform violence prevention efforts that address parental use of spanking. Therefore, the main aim of the current study was to determine if a wider range of parents’ ACEs history is associated with an increased likelihood of their child being spanked at age 10 or younger. 

## 2. Materials and Methods

### 2.1. Study Design and Participants

Data were drawn from the Well-Being and Experiences (WE) Study from Winnipeg, Manitoba and surrounding communities. The WE Study is an intergenerational longitudinal cohort program of research that involves multiple waves of data. At Wave 1, cross-sectional information was collected from parent/caregiver and adolescent pairs (*N* = 1000) between July 2017 and October 2018. Random digit dialing (21%), referrals (40.6%), and community advertisements (38.4%) were used to contact families with an adolescent aged 14 to 17 years. Adolescent demographic information was monitored to reflect sex, ethnicity, and household income distributions from the population which it was drawn [43]. A parent or caregiver most knowledgeable about the selected child (85% birth, step-, or adoptive mothers; 13% birth, step-, or adoptive fathers; 2% other caregivers; hereafter referred to as “parents”) completed a self-administered questionnaire at a research facility. Informed consent was obtained separately from both parent and adolescent participants. The WE Study was granted ethics approval by the Health Research Ethics Board at the University of Manitoba. The present study used data from Wave 1 of The WE Study, which included 1000 matched parents and adolescents. 

### 2.2. Measures

Spanking. Parents were asked to self-report whether the child who participated in the study was ever spanked by any parent or caregiver with their hand on the child’s bottom (bum) when the child was 10 years of age or younger with response options of “yes” or “no”.

Parent history of adverse childhood experiences (ACEs). Parents were asked to report on their history of an expanded list of 15 ACEs [39] before the age of 16 years: physical, sexual, and emotional abuse, physical and emotional neglect, spanking, exposure to physical IPV, household substance abuse, household mental illness, parental separation or divorce, parental trouble with police, parental gambling, contact with a CPO, poverty, and peer victimization (i.e., bullying). Physical abuse, sexual abuse, emotional abuse, physical neglect, and emotional neglect were measured using the Childhood Trauma Questionnaire (CTQ) and dichotomized following CTQ guidelines [44]. Spanking as an ACE was assessed with the question “In a typical year, when you were 10 years or younger, about how often do you remember an adult spanking you with their hand on your bottom (bum)?” Responses were coded as “yes” for spanking experienced “two to three times a year” or more often. All other responses (i.e., never, less than once a year, or once a year) were coded as “no” for spanking. We have used this approach of placing infrequent spanking in the no category in other publications [1,4]. The reason for this is that a parent or caregiver may spank a child once or only a few times and then choose not to do it again. Exposure to physical IPV was assessed with the question, “Before age 16, how many times did you see any one of your parents, step-parents or guardians hit each other or another adult in your home? By adult, we mean anyone 18 years and over.” and coded “yes” if the respondent witnessed this at least three times. Household substance abuse was assessed with two questions: (1) “Did a parent or other adult living in your home ever have problems with alcohol or spend a lot of time drinking or being hung over?” and (2) “Did a parent or other adult living in your home ever have problems with drugs?” and required an affirmative response to either one or both items to be coded “yes”. Household mental illness was assessed with the question “Did a parent or other adult living in your home ever have mental health issues like depression or anxiety?” (yes or no). Parental separation or divorce was assessed with the question “Were your biological parents ever separated or divorced?” (yes or no). Parental trouble with police was assessed with the question “Did a parent or other adult living in your home ever have problems with the police?” (yes or no). Parental gambling was assessed with the question “Did a parent or other adult living in your home ever have problems with gambling?” (yes or no). Contact with a CPO was assessed with the question “Did you ever see or talk to anyone from a child protective organization (like social services, child welfare, children’s aid, or the Ministry) due to difficulties at home?” (yes or no). As a proxy for poverty, two questions were asked: “How often did your family run out of money or find it hard to pay for…” (1) “rent or mortgage on your house?” and (2) “basic necessities like food or clothing?” and was coded “yes” if either one or both responses were “sometimes” or more often. Peer victimization was assessed using two experiences: (1) “Sometimes kids get hassled or picked on by other kids who say hurtful or mean things to them…” and (2) “Sometimes kids get physically pushed around, hit or beaten up by other kids or a group of kids. Before the age of 16, how many times did this happen to you?”; peer victimization was coded “yes” if the first item occurred “more than 10 times” and/or if the second occurred “3 to 5 times” or more often. Peer victimization definitions often require repeated behaviour toward another person [45,46,47]. To be consistent with this perspective and previous literature [43,48], peer victimization was coded in a similar manner for this work. 

Sociodemographic variables. Total household income received by all household members, from all sources, before taxes and deductions in the past 12 months was collected from the parent at Wave 1 and recoded into the following categories: CAD 49,999 or less, CAD 50,000 to CAD 99,999, CAD 100,000 to CAD 149,999, and CAD 150,000 or more. Parent age was recorded in years. Adolescent reported sex was assessed as male or female.

### 2.3. Statistical Analysis

First, descriptive statistics were computed for sociodemographic variables and parental ACEs by parent-reported use of spanking their child at age 10 years or younger. Second, logistic regression models were computed to examine the associations between parents’ history of individual ACEs and their self-reported history of their child being spanked in unadjusted models and models adjusting for parent age, child sex, and household income. 

## 3. Results

Characteristics of the sample stratified by parent-reported spanking of their child at age 10 years or younger are reported in Table 1. Parental age was associated with parent-reported use of spanking their child with increased parental age being related to decreased odds of spanking. Household income was also associated with parent-reported use of spanking with a household income of CAD 150,000 or more and no response to the income variable being associated with decreased odds of spanking compared to the lowest income of CAD 49,999 or less. No differences were reported for adolescent sex and likelihood of being spanked. Table 2 provides cross-tabulations and the associations between parents’ history of ACEs and parent-reported spanking of their own child. In unadjusted models, parents who had experienced physical abuse, emotional abuse, spanking, and household mental illness had increased odds that their own child would be spanked (Odd Ratios ranged from 1.40 to 2.28). In models adjusting for parent age, child sex, and household income, effect sizes were attenuated for physical abuse, emotional abuse, and spanking, but remained statistically associated with their child being spanked (Adjusted Odds Ratios ranged from 1.41 to 2.21).

## 4. Discussion

The main aim of the current study was to determine if a wider range of parents’ ACEs history is associated with an increased likelihood of their child being spanked at age 10 or younger. The current study advances knowledge with the inclusion of an expanded list of parental history of ACEs and likelihood that their own child would experience spanking. More specifically, findings indicate that parental history of experiencing physical abuse, emotional abuse, and spanking increases the likelihood that their child will be spanked. In addition, a child is more likely to be spanked if their own parent grew up in a household with a parent or other adult in the home experiencing mental health problems (unadjusted model only). Other parental household challenge ACEs including household substance use, parental separation or divorce, parental trouble with police, parental gambling, contact with CPO, poverty, and peer victimization were not associated with an increased likelihood that their own child would experience being spanked. However, it is possible that household mental illness in adjusted models and trouble with police, contact with CPO, sexual abuse, and peer victimization (unadjusted and adjusted models) may be underpowered and could represent Type II errors. These findings may indicate a potential Type II error related to limited power due to sample size. Future research to further understand how these early experiences of adversity may be related to spanking is warranted. 

The current study findings show both some consistencies and some inconsistencies with findings from another Canadian study. More specifically, consistent with the current work, data from parents and adolescents from Ontario, Canada indicated that a parent’s history of experiencing slapping/spanking and emotional abuse were related to an increased likelihood that their child or youth would be slapped/spanked, while parental physical neglect was not related to children’s experiences of spanking in either study [42]. Importantly, parental experiences of physical abuse demonstrated reverse relationships in each study with the current study finding an increased likelihood with their child experiencing spanking and the Ontario data indicating decreased likelihood with their child experiencing slapping/spanking [42]. It is possible that growing up in a violent household may lead to a tolerance of violence and the continued use of physical force across generations. It is also the possible that exposure to violence may lead to perpetration of violence as it may seem normative or how to solve or deal with problems. This theoretical perspective would support the findings for the current study. However, it is also possible that some people who are physically abused do not want their own children to experience the same violence inflicted upon them, and purposefully avoid using physical force including spanking [49]. More work is needed to further clarify this relationship including identifying underlying mechanisms that may help to inform the development of violence prevention efforts that specifically target parental use of spanking and other forms of punishment. Data from Ontario also found that parental exposure to physical IPV, sexual abuse, and peer victimization were associated with an increased likelihood their child or youth would be spanked [42], which were found to be non-significant in the current study. 

The limitations of the current study should be considered when interpreting the study’s findings. First, the data are cross-sectional in nature meaning inferences about causation are not possible. Second, data on ACEs are retrospective and may be subject to recall bias. However, it is important to note that previous evidence does show that retrospective recall of childhood adversity provides valid and reliable findings [50,51,52]. Third, due to social desirability, some parents or caregivers may be reluctant to report spanking their child. Reducing concerns of social desirability is one reason why parents were asked to self-report whether the child who participated in the study was ever spanked by any parent or caregiver with their hand on the child’s bottom (bum). Our findings indicated that approximately 50% of male and female adolescents were spanked, indicating that social desirability was not likely a problem in this study. Fourth, the relationship of the person spanking the child is unknown and may not be the parent respondent in this study. It is possible that a parent was not aware of spanking by another caregiver, indicating that the current findings may actually be more conservative. Finally, it is possible that some models were underpowered, as indicated by medium effect sizes and wider and non-significant confidence intervals. These models should be interpreted with caution and included in future research using a large sample size. In addition, it may be informative for future research using a larger sample size to possibly include interaction effects between ACEs and sociodemographic variables and possible dose–response relationships. However, future research examining individual ACEs should be a priority as it provides more information from an intervention perspective compared to an ACEs count variable. 

## 5. Conclusions

Based on current knowledge, it has been recommended that children and adolescents should never be spanked [1,13,14]. Few violence prevention programs that specifically target parental use of physical punishment are available, with emerging and varying levels of evidence of effectiveness [53]. Understanding and examining parental ACEs history, specifically physical abuse, emotional abuse, spanking, and household mental illness, may help to identify families who may be more likely to use physical punishment with their children and may be an important area of consideration when informing efforts to prevent spanking. Early identification of at-risk behaviours is a critical aspect of prevention and should be a global public health priority. This is an important goal since preventing violence against children including spanking, is necessary to optimize healthy child development, reduce the risk of further violence and intergenerational transmission of family violence, and advance children’s right to protection. 

## Figures and Tables

**Table 1:** Sociodemographic characteristics of the sample stratified by parent-reported spanking.

	Parent-Reported Use of Spanking Their Child, Age 10 or Younger		
	No	Yes	OR (95% CI)
Parent age, years	Mean (sd)	Mean (sd)	
	45.93 (5.96)	44.19 (5.81)	0.95 (0.93–0.97) ***
	% (n)	% (n)	
Household income			
CAD 49,999 or less (reference)	18.8 (109)	21.1 (80)	1.00
CAD 50,000 to CAD 99,999	32.0 (185)	39.5 (150)	1.10 (0.77–1.58)
CAD 100,000 to CAD 149,999	22.6 (131)	22.4 (85)	0.88 (0.59–1.32)
CAD 150,000 or more	20.4 (118)	14.7 (56)	0.65 (0.42–0.99) *
No response	6.2 (36)	2.4 (9)	0.34 (0.16–0.75) **
Adolescent sex			
Male (reference)	46.7 (270)	50.5 (191)	1.00
Female	53.3 (308)	49.5 (187)	0.86 (0.66–1.11)

Note: OR = odds ratio; CI = confidence interval. * *p* ≤ 0.05; ** *p* ≤ 0.01; *** *p* ≤ 0.001.

**Table 2:** Associations between parents’ history of ACEs and parent-reported spanking.

Parent History of ACEs	Parent-Reported Use of Spanking Their Child, Age 10 or Younger			
	No % (n)	Yes % (n)	OR (95% CI)	AOR (95% CI)
Physical abuse	19.9 (115)	26.3 (99)	1.44 (1.06–1.96) *	1.41 (1.02–1.93) *
Sexual abuse	24.9 (141)	29.4 (108)	1.26 (0.94–1.69)	1.23 (0.90–1.66)
Emotional abuse	14.6 (84)	21.1 (79)	1.56 (1.11–2.19) **	1.43 (1.01–2.04) *
Physical neglect	25.8 (149)	23.2 (88)	0.87 (0.64–1.18)	0.81 (0.60–1.11)
Emotional neglect	14.3 (82)	14.0 (52)	0.98 (0.67–1.42)	0.94 (0.64–1.39)
Spanking	37.5 (215)	57.8 (215)	2.28 (1.75–2.98) ***	2.21 (1.68–2.90) ***
Exposure to physical IPV	11.7 (67)	13.6 (51)	1.19 (0.80–1.75)	1.07 (0.71–1.60)
Household substance abuse	30.0 (170)	31.1 (116)	1.05 (0.79–1.40)	0.97 (0.73–1.30)
Household mental illness	29.9 (156)	37.5 (130)	1.40 (1.05–1.87) *	1.31 (0.98–1.76)
Parental separation or divorce	22.4 (125)	25.7 (93)	1.20 (0.88–1.63)	1.09 (0.79–1.50)
Parental trouble with police	7.2 (41)	10.8 (40)	1.55 (0.98–2.45)	1.30 (0.81–2.08)
Parental gambling	6.0 (34)	6.5 (24)	1.09 (0.64–1.87)	0.91 (0.52–1.58)
Contact with a CPO	6.5 (37)	9.0 (34)	1.43 (0.88–2.32)	1.17 (0.70–1.95)
Poverty	37.6 (205)	42.7 (149)	1.24 (0.94–1.63)	1.05 (0.79–1.40)
Peer victimization	43.1 (242)	48.9 (182)	1.26 (0.97–1.64)	1.26 (0.96–1.65)

Note: ACE = adverse childhood experiences; OR = odds ratio; AOR = odds ratio adjusted for parent age, child sex, and household income; CI = confidence interval; CPO = child protective organization; IPV = intimate partner violence. * *p* ≤ 0.05; ** *p* ≤ 0.01; *** *p* ≤ 0.001.

## Data Availability

Due to health ethics research board guidelines, the data are not publicly available.

## References

[1] Afifi T.O., Ford D., Gershoff E.T., Merrick M., Grogan-Kaylor A., Ports K.A., MacMillan H.L., Holden G.W., Taylor C.A., Lee S.J. (2017). Spanking and adult mental health impairment: The case for the designation of spanking as an adverse childhood experience. Child Abus. Negl..

[2] Afifi T.O., Mota N.P., Dasiewicz P., MacMillan H.L., Sareen J. (2012). Physical punishment and mental disorders: Results from a nationally representative US sample. Pediatrics.

[3] Afifi T.O., Mota N., MacMillan H.L., Sareen J. (2013). Harsh physical punishment in childhood and adult physical health. Pediatrics.

[4] Fortier J., Stewart-Tufescu A., Salmon S., MacMillan H.L., Gonzalez A., Kimber M., Duncan L., Taillieu T., Garces Davila I., Struck S. (2021). Associations between lifetime spanking/slapping and adolescent physical and mental health and behavioral outcomes. Can. J. Psychiatry.

[5] Merrick M.T., Ports K.A., Ford D.C., Afifi T.O., Gershoff E.T., Grogan-Kaylor A. (2017). Unpacking the impact of adverse childhood experiences on adult mental health. Child Abus. Negl..

[6] Altschul I., Lee S.J., Gershoff E.T. (2016). Hugs, not hits: Warmth and spanking as predictors of child social competence. J. Marriage Fam..

[7] Gershoff E.T., Grogan-Kaylor A. (2016). Spanking and child outcomes: Old controversies and new meta-analyses. J. Fam. Psychol..

[8] Gershoff E.T. (2013). Spanking and child development: We know enough now to stop hitting our children. Child Dev. Perspect..

[9] Lee S.J., Taylor C.A., Altschul I., Rice J.C. (2013). Parental spanking and subsequent risk for child aggression in father-involved families of young children. Child. Youth Serv. Rev..

[10] Gershoff E.T. (2002). Corporal punishment by parents and associated child behaviors and experiences: A meta-analytic and theoretical review. Psychol. Bull..

[11] Committee on Psychosocial Aspects of Child and Family Health (1998). Guidance for effective discipline. Pediatrics.

[12] Heilmann A., Mehay A., Watt R.G., Kelly Y., Durrant J.E., van Turnhout J., Gershoff E.T. (2021). Physical punishment and child outcomes: A narrative review of prospective studies. Lancet.

[13] Durrant J., Ensom R. (2012). Physical punishment of children: Lessons from 20 years of research. CMAJ.

[14] Sege R.D., Siegel B.S., AAP Council on Child Abuse and Neglect, AAP Committee on Psychosocial Aspects of Child and Family Health (2018). Effective discipline to raise healthy children. Pediatrics.

[15] Williams R.C., Biscaro A., Clinton J., Canadian Paediatric Society Early Years Task Force Relationships Matter: How Clinicians Can Support Positive Parenting in the Early Years. https://www.cps.ca/en/documents/position/positive-parenting.

[16] World Health Organization Corporal Punishment and Health. https://www.who.int/news-room/fact-sheets/detail/corporal-punishment-and-health.

[17] Durrant J.E., Ensom R., Coalition on Physical Punishment of Children and Youth (2004). Joint Statement on Physical Punishment of Children and Youth.

[18] Afifi T.O., Salmon S., Stewart-Tufescu A., Taillieu T., Fortier J., MacMillan H., Durrant J., Holden G.W. (2022). Associations between spanking beliefs and reported spanking among adolescents-parent/caregiver dyads in a Canadian sample. BMC Public Health.

[19] Clément M., Chamberland C. (2014). Trends in corporal punishment and attitudes in favour of this practice: Toward a change in societal norms. Can. J. Community Ment. Health.

[20] Cuartas J., McCoy D.C., Rey-Guerra C., Britto P.R., Beatriz E., Salhi C. (2019). Early childhood exposure to non-violent discipline and physical and psychological aggression in low- and middle-income countries: National, regional, and global prevalence estimates. Child Abus. Negl..

[21] Straus M.A. (2010). Prevalence, societal causes, and trends in corporal punishment by parents in world perspective. Law Contemp. Probl..

[22] Zolotor A.J., Theodore A.D., Runyan D.K., Chang J.J., Laskey A.L. (2011). Corporal punishment and physical abuse: Population-based trends for three-to-11-year-old children in the United States. Child Abus. Rev..

[23] Lansford J.E., Cappa C., Putnick D.L., Bornstein M.H., Deater-Deckard K., Bradley R.H. (2017). Change over time in parents’ beliefs about and reported use of corporal punishment in eight countries with and without legal bans. Child Abus. Negl..

[24] Finkelhor D., Turner H., Wormuth B.K., Vanderminden J., Hamby S. (2019). Corporal punishment: Current rates from a national survey. J. Child Fam. Stud..

[25] Taillieu T.L., Afifi T.O., Mota N., Keyes K.M., Sareen J. (2014). Age, sex, and racial differences in harsh physical punishment: Results from a nationally representative United States sample. Child Abus. Negl..

[26] United Nations General Assembly (1989). Convention on the Rights of the Child. Treaty Series..

[27] United Nations Committee on the Rights of the Child (2007). General Comment No. 8 (2006). The Right of the Child to Protection from Corporal Punishment and other Cruel or Degrading forms of Punishment (CRC/C/GC/8), Para 3.

[28] United Nations Sustainable Development Knowledge Platform. https://sustainabledevelopment.un.org/sdgs.

[29] Global Initiative to End All Corporal Punishment of Children Global Progress: Countdown to Universal Prohibition. https://endcorporalpunishment.org/countdown/.

[30] Madigan S., Cyr C., Eirich R., Fearon R.M.P., Ly A., Rash C., Poole J.C., Alink L.R.A. (2019). Testing the cycle of maltreatment hypothesis: Meta-analytic evidence of the intergenerational transmission of child maltreatment. Dev. Psychopathol..

[31] Deater-Deckard K., Lansford J.E., Dodge K.A., Pettit G.S., Bates J.E. (2003). The development of attitudes about physical punishment: An 8-year longitudinal study. J. Fam. Psychol..

[32] Lunkenheimer E.S., Kittler J.E., Olson S.L., Kleinberg F. (2006). The intergenerational transmission of physical punishment: Differing mechanisms in mothers’ and fathers’ endorsement?. J. Fam. Violence.

[33] Markowitz F.E. (2001). Attitudes and family violence: Linking intergenerational and cultural theories. J. Fam. Violence.

[34] Simons D.A., Wurtele S.K. (2010). Relationships between parents’ use of corporal punishment and their children’s endorsement of spanking and hitting other children. Child Abus. Negl..

[35] Clément M., Chamberland C. (2009). The role of parental stress, mother’s childhood abuse and perceived consequences of violence in predicting attitudes and attribution in favor of corporal punishment. J. Child Fam. Stud..

[36] Chung E.K., Mathew L., Rothkopf A.C., Elo I.T., Coyne J.C., Culhane J.F. (2009). Parenting attitudes and infant spanking: The influence of childhood experiences. Pediatrics.

[37] Gagne M., Tourigny M., Joly J., Pouliot-Lapointe J. (2007). Predictors of adult attitudes toward corporal punishment of children. J. Interpers. Violence.

[38] Felitti V.J., Anda R.F., Nordenberg D., Williamson D.F., Spitz A.M., Edwards V., Koss M.P., Marks J.S. (1998). Relationship of childhood abuse and household dysfunction to many of the leading causes of death in adults: The Adverse Childhood Experiences (ACE) Study. Am. J. Prev. Med..

[39] Afifi T.O., Salmon S., Garces I., Struck S., Fortier J., Taillieu T., Stewart-Tufescu A., Asmundson G.J.G., Sareen J., MacMillan H.L. (2020). Confirmatory factor analysis of adverse childhood experiences (ACEs) among a community-based sample of parents and adolescents. BMC Pediatr..

[40] Buffarini R., Hammerton G., Coll C.V.N., Cruz S., da Silveira M.F., Murray J. (2022). Maternal adverse childhood experiences (ACEs) and their associations with intimate partner violence and child maltreatment: Results from a Brazilian birth cohort. Prev. Med..

[41] Yoon Y., Cederbaum J.A., Mennen F.E., Traube D.E., Chou C.P., Lee J.O. (2019). Linkage between teen mother’s childhood adversity and externalizing behaviors in their children at age 11: Three aspects of parenting. Child Abus. Negl..

[42] Afifi T.O., Fortier J., MacMillan H.L., Gonzalez A., Kimber M., Georgiades K., Duncan L., Taillieu T., Davila I.G., Struck S. (2019). Examining the relationships between parent experiences and youth self-reports of slapping/spanking: A population-based cross-sectional study. BMC Public Health.

[43] Afifi T.O., Taillieu T., Salmon S., Davila I.G., Stewart-Tufescu A., Fortier J., Struck S., Asmundson G.J.G., Sareen J., MacMillan H.L. (2020). Adverse childhood experiences (ACEs), peer victimization, and substance use among adolescents. Child Abus. Negl..

[44] Bernstein D.P., Fink L. (1998). Childhood Trauma Questionnaire: A Retrospective Self-Report.

[45] Freeman J.G., King M., Pickett W. (2011). The Health of Canada’s Young People: A Mental Health Focus.

[46] Gladden R.M., Vivolo-Kantor A.M., Hamburger M.E., Lumpkin C.D. (2014). Bullying Surveillance among Youths: Uniform Definitions for Public Health and Recommended Data Elements, Version 1.0.

[47] Olweus D. (1996). Revised Olweus Bully/Victim Questionnaire.

[48] Salmon S., Garces Davila I., Taillieu T.L., Stewart-Tufescu A., Duncan L., Fortier J., Struck S., Georgiades K., MacMillan H.L., Kimber M. (2022). Adolescent health outcomes: Associations with child maltreatment and peer victimization. BMC Public Health.

[49] Ertem I.O., Leventhal J.M., Dobbs S. (2000). Intergenerational continuity of child physical abuse: How good is the evidence?. Lancet.

[50] Hardt J., Vellaisamy P., Schoon I. (2010). Sequelae of prospective versus retrospective reports of adverse childhood experiences. Psychol. Rep..

[51] Hardt J., Rutter M. (2004). Validity of adult retrospective reports of adverse childhood experiences: Review of the evidence. J. Child Psychol. Psychiatry.

[52] Hardt J., Sidor A., Bracko M., Egle U.T. (2006). Reliability of retrospective assessments of childhood experiences in Germany. J. Nerv. Ment. Dis..

[53] Gershoff E.T., Lee S.J., Durrant J.E. (2017). Promising intervention strategies to reduce parents’ use of physical punishment. Child Abus. Negl..

